# Supplementary material for: Genus-wide genomic characterization of Macrococcus: insights into evolution, population structure, and functional potential
Source: Front Microbiol. 2023 Jul 20;14:1181376. doi: 10.3389/fmicb.2023.1181376 (PMC10400458; doi:10.3389/fmicb.2023.1181376)
Supplement: Supplementary file 2 [file Image_1.PDF]

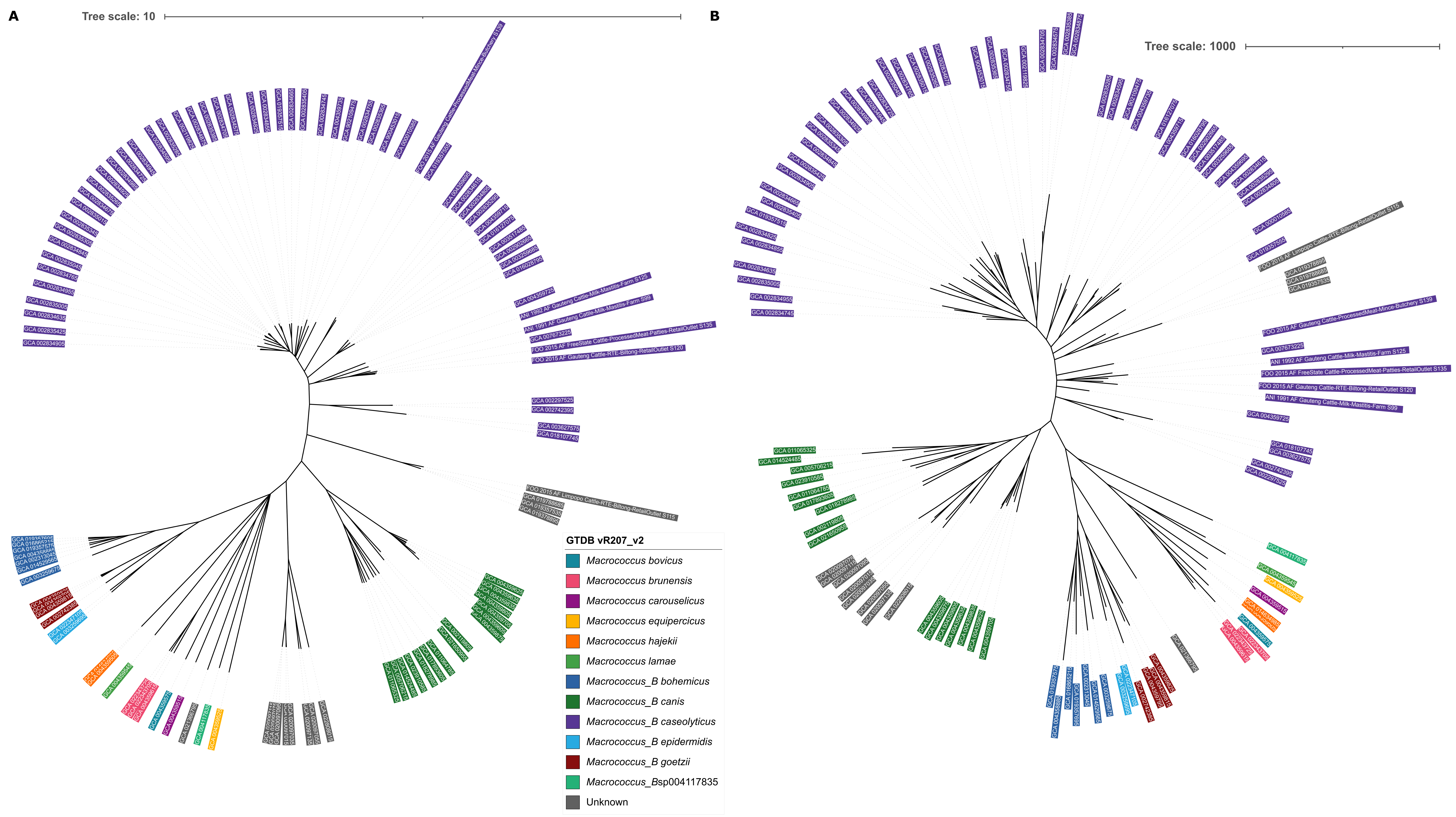

Supplementary Figure S1. Unrooted (A) Core Genome Allelic Variation (CGAV) tree and (B) accessory gene presence/absence tree, constructed using PEPPAN, a 40% amino acid identity threshold, and a 95% core genome threshold (n = 110 Macrococcus genomes). Tip label colors correspond to Genome Taxonomy Database (GTDB) species, assigned using the Genome Taxonomy Database Toolkit (GTDB-Tk) v2.1.0 and GTDB vR207_v2. PEPPAN constructed the (A) CGAV tree using RapidNJ based on numbers of identical sequences (i.e., alleles) of single copy genes present in ≥95% of Macrococcus genomes. For (B), PEPPAN used FastTree to construct the tree, using the binary presence/absence of accessory genes.
