# Supplementary figures and images for: Genus-wide genomic characterization of Macrococcus: insights into evolution, population structure, and functional potential
Source: Front Microbiol. 2023 Jul 20;14:1181376. doi: 10.3389/fmicb.2023.1181376 (PMC10400458; doi:10.3389/fmicb.2023.1181376)

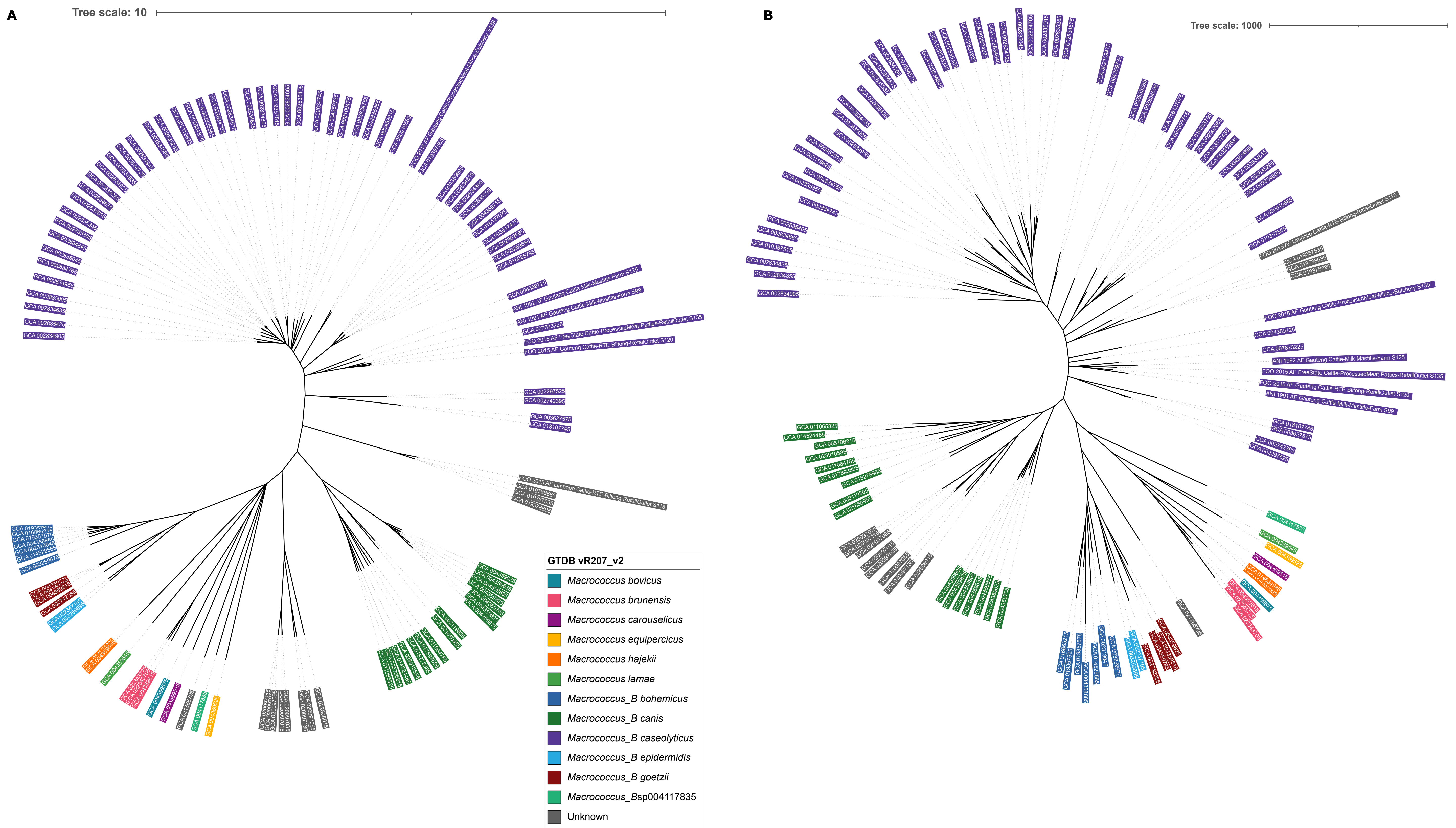

Supplement: Supplementary file 3 [file Image_2.PDF]

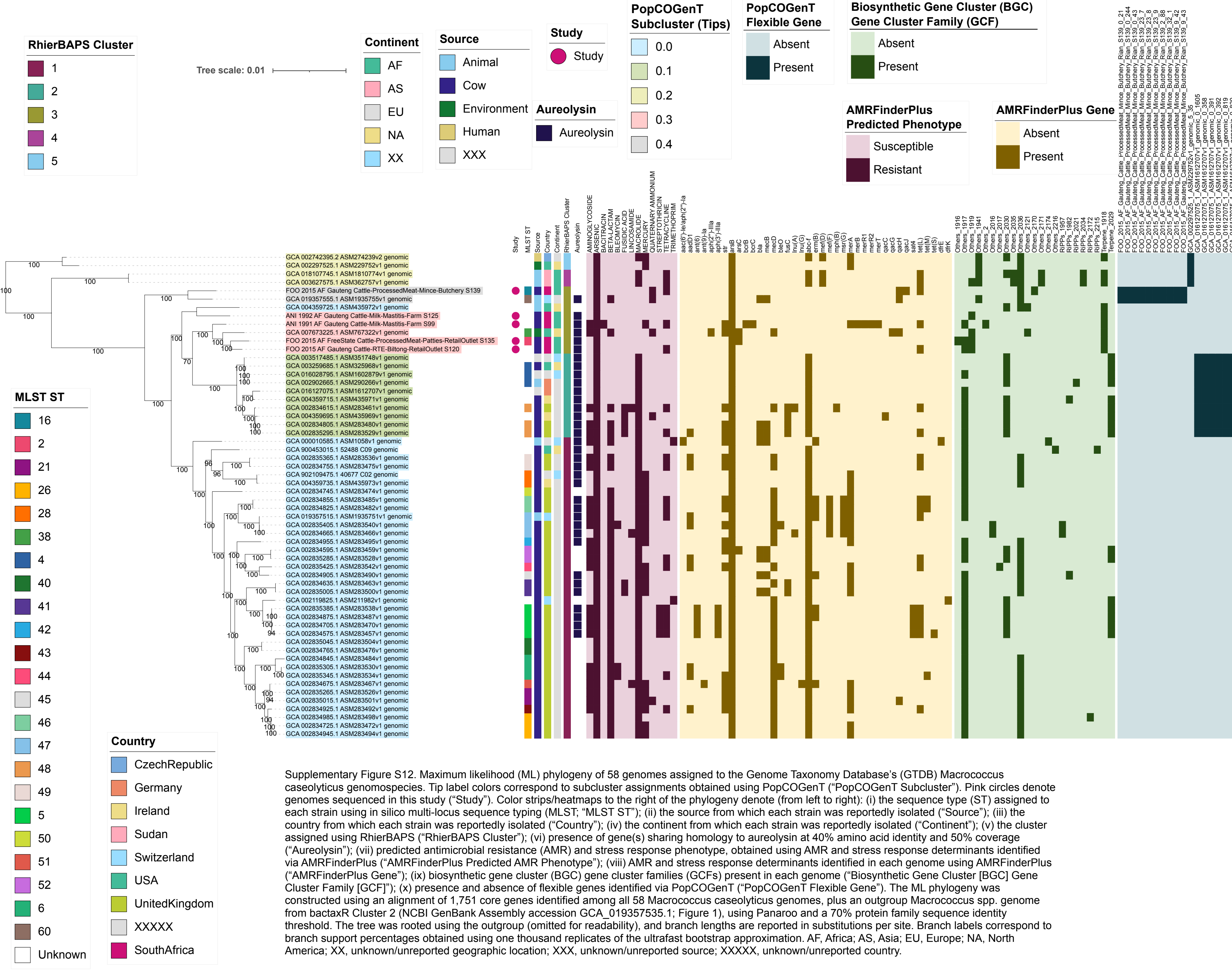

Supplement: Supplementary file 13 [file Image_12.PDF]

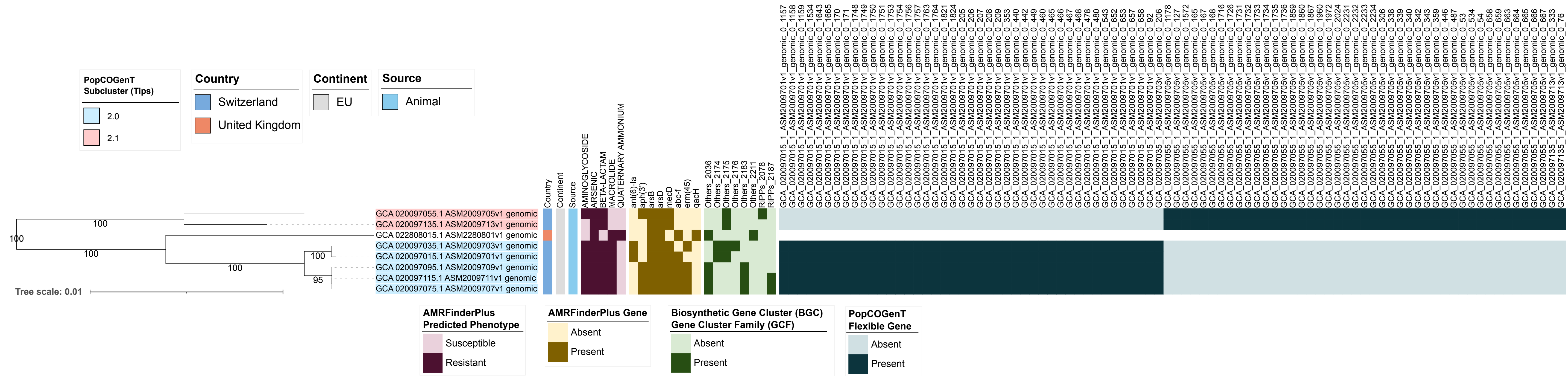

Supplement: Supplementary file 14 [file Image_13.PDF]
