# Supplementary material for: Genus-wide genomic characterization of Macrococcus: insights into evolution, population structure, and functional potential
Source: Front Microbiol. 2023 Jul 20;14:1181376. doi: 10.3389/fmicb.2023.1181376 (PMC10400458; doi:10.3389/fmicb.2023.1181376)
Supplement: Supplementary file 5 [file Image_4.PDF]

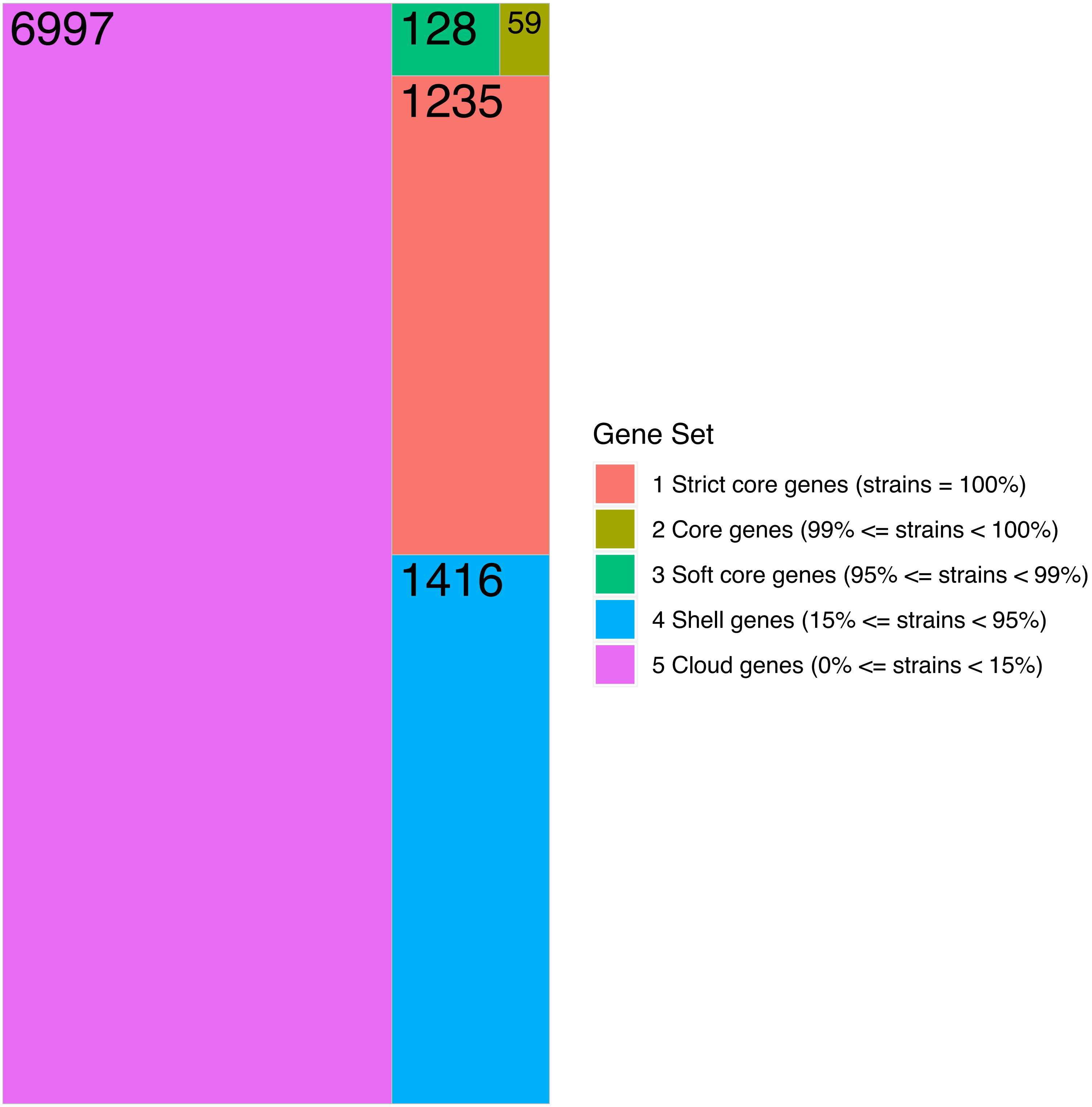

Supplementary Figure S4. Treemap showcasing the number of genes detected within a given percentage of Macrococcus genomes (out of 110 total genomes). Tile sizes are proportional to the number of genes detected within a given percentage of Macrococcus genomes; numerical labels within each tile denote the corresponding number of genes. PEPPAN was used to construct the core- and pan-genomes using a 20% amino acid identity threshold and a core genome threshold of 95%
