# Supplementary material for: Genus-wide genomic characterization of Macrococcus: insights into evolution, population structure, and functional potential
Source: Front Microbiol. 2023 Jul 20;14:1181376. doi: 10.3389/fmicb.2023.1181376 (PMC10400458; doi:10.3389/fmicb.2023.1181376)
Supplement: Supplementary file 9 [file Image_8.PDF]

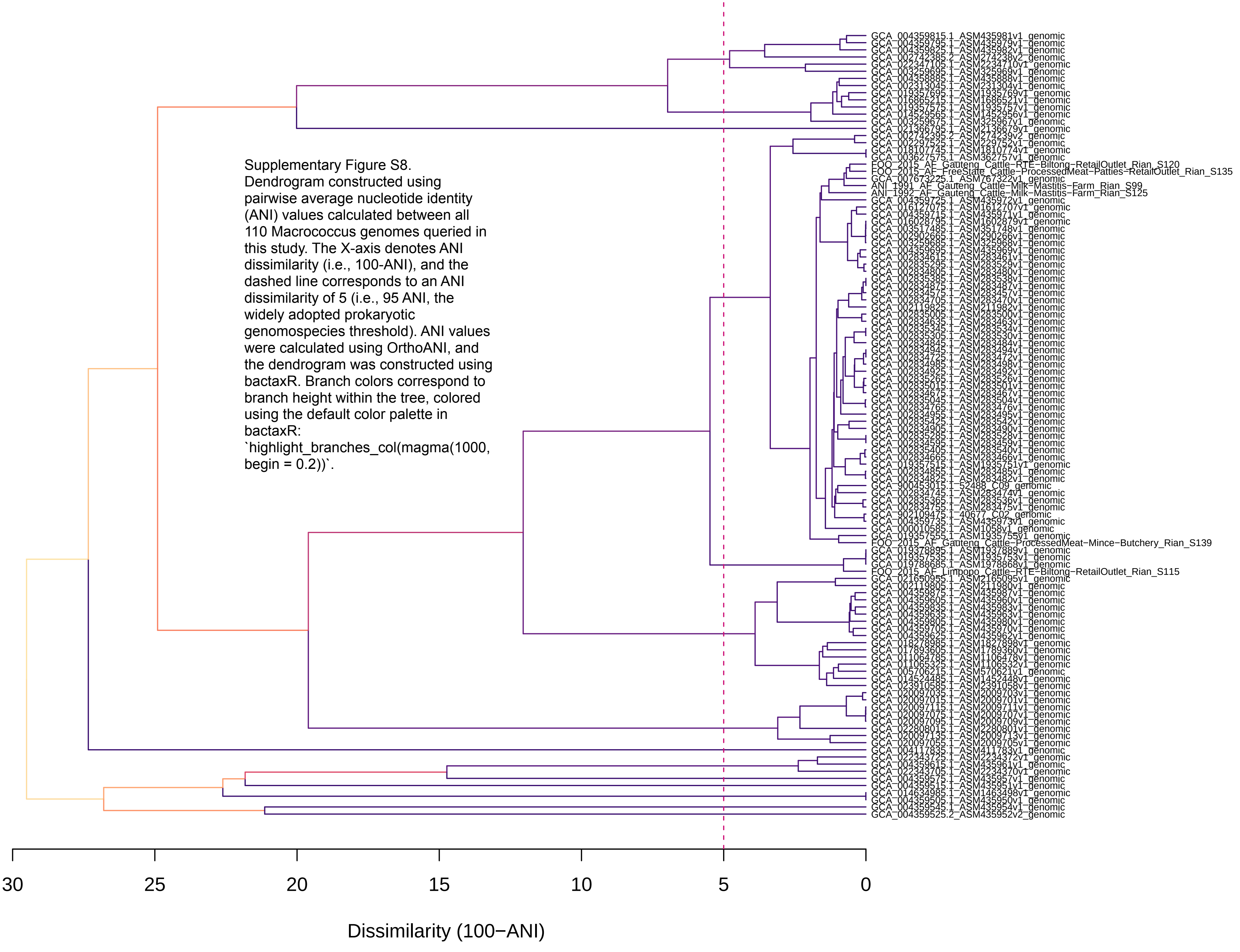

Supplementary Figure S8. Dendrogram constructed using pairwise average nucleotide identity (ANI) values calculated between all 110 Macrococcus genomes queried in this study. The X-axis denotes ANI dissimilarity (i.e., 100-ANI), and the dashed line corresponds to an ANI dissimilarity of 5 (i.e., 95 ANI, the widely adopted prokaryotic genomospecies threshold). ANI values were calculated using OrthoANI, and the dendrogram was constructed using bactaxR. Branch colors correspond to branch height within the tree, colored using the default color palette in bactaxR: `highlight_branches_col(magma(1000, begin = 0.2))`.
