# Supplementary material for: Genus-wide genomic characterization of Macrococcus: insights into evolution, population structure, and functional potential
Source: Front Microbiol. 2023 Jul 20;14:1181376. doi: 10.3389/fmicb.2023.1181376 (PMC10400458; doi:10.3389/fmicb.2023.1181376)

Supplementary Figure S10. Maximum likelihood (ML) phylogeny of all 104 high-quality, publicly available Macrococcus genomes, plus six bovine-associated South African genomes sequenced here (n = 110 total Macrococcus genomes). Tip label colors correspond to genomospecies assignments obtained via the Genome Taxonomy Database Toolkit (GTDB-Tk) v2.1.0 and GTDB vR207_v2. Genomes of strains isolated and sequenced in this study are denoted by pink circles ("Study"). Color strips and heatmaps to the right of the phylogeny denote (from left to right): (i) the source from which each strain was reportedly isolated ("Source"); (ii) the continent from which each strain was reportedly isolated ("Continent"); (iii) antimicrobial resistance (AMR) and stress response determinants identified in each genome using AMRFinderPlus (default settings; "AMRFinderPlus Determinant"). The ML phylogeny was constructed using an alignment of 649 core genes identified among all 110 Macrococcus genomes, plus the genome of Staphylococcus aureus str. DSM 20231 (outgroup genome; NCBI RefSeq Assembly accession GCF_001027105.1), using Panaroo and a 50% protein family sequence identity threshold. The tree was rooted using the outgroup (omitted for readability), and branch lengths are reported in substitutions per site. AF, Africa; AS, Asia; EU, Europe; NA, North America; XX, unknown/unreported geographic location.

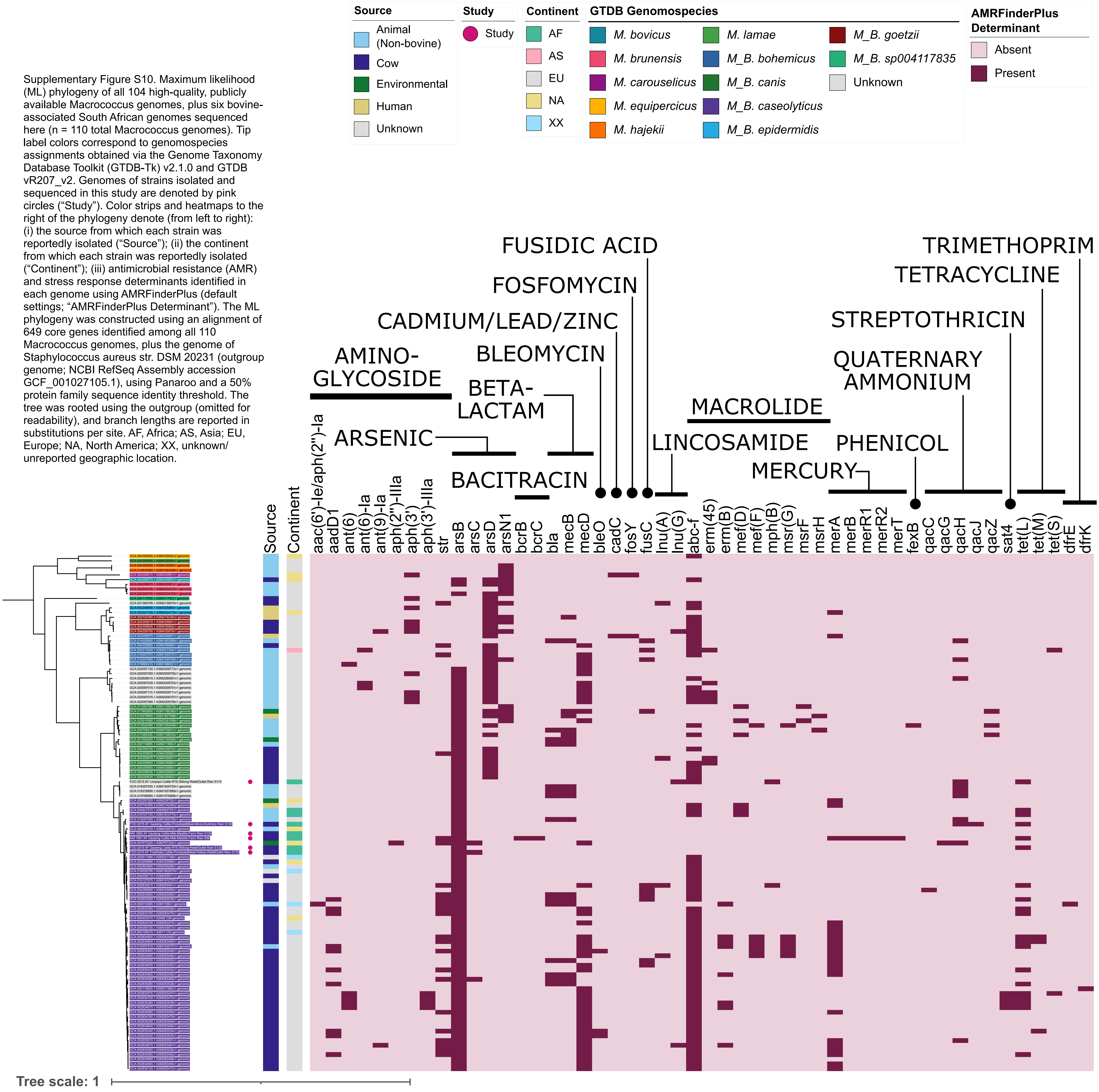

Supplement: Supplementary file 11 [file Image_10.PDF]
